# Abatacept with methotrexate versus other biologic agents in treatment of patients with active rheumatoid arthritis despite methotrexate: a network meta-analysis

**DOI:** 10.1186/ar3537

**Published:** 2011-12-12

**Authors:** Patricia Guyot, Peter Taylor, Robin Christensen, Louisa Pericleous, Coralie Poncet, Maximilian Lebmeier, Pieter Drost, Gert Bergman

**Affiliations:** 1 Mapi Values the Netherlands, De Molen 84, 3995 AX Houten, The Netherlands; 2 Kennedy Institute of Rheumatology Division, Imperial College, 65 Aspenlea Road, Hammersmith, London, W6 8LH, UK; 3 Musculoskeletal Statistics Unit (MSU), The Parker Institute, Copenhagen University Hospital at Frederiksberg, Nordre Fasanvej 57, DK-2000 Copenhagen F, Denmark; 4 Bristol-Myers Squibb Pharmaceuticals Ltd., Uxbridge Business Park, Sanderson Road, Uxbridge, Middlesex, UB8 1DH, UK; 5 DOCS International, 20 rue Troyon, 92310 Sèvres, France; 6 Bristol-Myers Squibb International Corporation, Avenue de Finlande 8, 1420 Braine l' Alleud, Belgium

**Keywords:** abatacept, rheumatoid arthritis, biologic DMARDs, network meta-analysis, health assessment questionnaire

## Abstract

**Introduction:**

The goal of this study was to compare the efficacy in terms of Health Assessment Questionnaire change from baseline (HAQ CFB), 50% improvement in American College of Rheumatology criterion (ACR-50) and Disease Activity Score in 28 joints (DAS28) defined remission (< 2.6) between abatacept and other biologic disease modifying anti-rheumatic drugs (DMARDs) in patients with rheumatoid arthritis (RA) who have inadequate response to methotrexate (MTX-IR).

**Methods:**

A systematic literature review identified controlled trials investigating the efficacy of abatacept (three studies), etanercept (two studies), infliximab (two), adalimumab (two), certolizumab pegol (two) ritixumab (three), and tocilizumab (two) in MTX-IR patients with RA. The clinical trials included in this analysis were similar with respect to trial design, baseline patient characteristics and background therapy (MTX). The key clinical endpoints of interest were HAQ CFB, ACR-50 and DAS28 < 2.6 measured at 24 and 52 weeks. The results were analysed using network meta-analysis methods that enabled calculation of an estimate for expected relative effect of comparative treatments. Analysis results were expressed as the difference in HAQ CFB score and odds ratio (OR) of achieving an ACR-50 and DAS28 response and associated 95% credible intervals (CrI).

**Results:**

The analysis of HAQ CFB at 24 weeks and 52 weeks showed that abatacept in combination with MTX is expected to be more efficacious than MTX monotherapy and is expected to show a comparable efficacy relative to other biologic DMARDs in combination with MTX. Further, abatacept showed comparable ACR-50 and DAS28 < 2.6 response rates with other biologic DMARDs at 24 and 52 weeks, except for ACR-50 compared to certolizumab pegol at 52 weeks and for DAS28 < 2.6 compared to tocilizumab at 24 weeks. Sensitivity analyses confirmed the robustness of the findings.

**Conclusions:**

Abatacept in combination with MTX is expected to result in a comparable change from baseline in HAQ score and comparable ACR-50 and DAS28 < 2.6 response rates in MTX-IR patients compared to other approved biologic agents.

## Introduction

Rheumatoid arthritis (RA) is a chronic, disabling systemic inflammatory disorder, with immune-mediated attacks of the synovial joints. Disease-modifying anti-rheumatic drugs (DMARDs) alleviate the symptoms of RA and have the potential to slow or stop disease progression [1-3]. DMARDs are classified into two types: conventional and biologic. European Guidelines recommend that methotrexate (MTX), a conventional DMARD, is included in the first-line treatment strategy for active RA as soon as possible after diagnosis [4]. In patients with an insufficient response to treatment with MTX and/or other conventional DMARDs, biologic DMARDs designed to target specific elements of the immune system involved in the inflammation and damage to joints should be combined with MTX to improve the outcome, in particular TNF inhibitors [4]. Currently licensed TNF inhibitors for patients with RA showing active disease despite MTX therapy include infliximab [5], etanercept [6], adalimumab [7], certolizumab pegol [8] and golimumab [9]. Other licensed biologic agents with alternative mechanisms of action include tocilizumab [10] and abatacept [11]; also rituximab [12] was under evaluation for approval in this patient population at the time of this analysis.

Abatacept is the first in class of biologic DMARDs and acts by selectively modulating an essential co-stimulatory pathway needed for T-cell activation, thus inhibiting the inflammatory process upstream in the cascade of inflammatory events of importance in the pathology of RA [13]. The effectiveness of abatacept has been demonstrated in a series of randomised controlled trials [14-18]. Ideally, in order that decisions on treatment options could be made based on firm clinical evidence, the comparative efficacy of each and every treatment option would be known. However, given the lack of head-to-head data for direct comparison, network meta-analyses are necessary in order to calculate the expected efficacy of biologic DMARDs. Indirect comparisons of interventions can be made through a common comparator [19].

Our objective was to perform a network meta-analysis for abatacept following a systematic review of the published clinical evidence of abatacept and all other existing biologic DMARDs available, licensed in Europe for patients that failed to respond to MTX or in the process of obtaining such a license. The aim of the study was to estimate the relative efficacy of abatacept in combination with MTX in Health Assessment Questionnaire change from baseline (HAQ score CFB) compared to other relevant biologic DMARDs plus MTX in the treatment of patients with RA with insufficient response to MTX. As a secondary aim, we studied the efficacy in terms of response rates of the American College Rheumatology Criterion for 50% improvement (ACR-50) and in Disease Activity Score in 28 joints (DAS28) defined remission (< 2.6).

## Materials and methods

### Systematic review

A protocol was developed to define the search strategy and a systematic review performed consecutively to identify those randomised controlled trials (RCTs), which investigated the efficacy of biologic DMARDs licensed to treat RA with insufficient response to at least one conventional DMARD. MEDLINE and EMBASE databases were searched simultaneously using Datastar. Further searches were undertaken for the Cochrane Library, the American College of Rheumatology (ACR) and European League Against Rheumatism (EULAR) conferences, and the technology appraisals for the UK. Searches included a combination of free-text and Medline Subject Headings (MeSH) terms for 'disease terms' with 'drug names', and were limited to 'human' RCTs published, in English, between January 1980 and January 2010.

The systematic review was performed by two researchers, with discussions between the two to come to agreement in case of discrepancies. The full-text articles were assessed for inclusion according to the following selection criteria: (1) treatment combinations of MTX with abatacept, adalimumab, certolizumab, etanercept, golimumab, infliximab, rituximab or tocilizumab in comparison with each other or Placebo + MTX; (2) RA patients with an inadequate response or intolerance to previous treatment with at least one conventional DMARD (MTX, sulfasalazine, leflunomide, azathioprine, gold salts or minocycline); (3) clinical endpoints of HAQ CFB [20,21], American College of Rheumatology Criterion of 50% improvement (ACR-50) [22] and remission defined by a Disease Activity Score including a 28-joint count less than 2.6 (DAS28 < 2.6) [23]; at 24 and/or 52 weeks.

### Data collection

For each selected study, the details of design, selection criteria, study population characteristics, interventions, outcome measures, length of follow-up and results were extracted and recorded in data extraction forms. The data extraction was performed by one researcher and reviewed by another; meaning, effectively, that the second reviewer traced back every value/number/comment to the original full text report and validated the extracted data.

### Network meta-analyses

The search strategy was developed in order to capture all the relevant studies; but to ensure more coherent network meta-analyses, the inclusion criteria used for the analyses were restricted as follows: (1) only recommended dosages licensed for treatment in Europe [5-12] and (2) only RA patients with an inadequate response or intolerance to MTX. The quantitative results of the different interventions from the studies identified were combined using Bayesian mixed treatment comparison techniques [19]. All analyses were performed using a non-informative prior distribution and, depending on the heterogeneity as assessed by the goodness-of-fit test based on the residual deviance [19], either a fixed effect or a random effects model was chosen. Analyses were performed for the endpoints of HAQ CFB (continuous outcome), ACR-50 and DAS28 < 2.6 response rates (dichotomous outcomes) using placebo (in combination with MTX) as the common comparator. The network meta-analysis results present estimates of the differences in mean HAQ CFB, and estimates of odds ratio (OR) for ACR-50 and DAS28 < 2.6, for each biologic agent compared with placebo and for each pairwise combination of biologic agents. By using the average absolute placebo response (calculated as the weighted mean placebo response based on all included trials) as a baseline, the relative efficacy of each treatment compared with placebo was adjusted to obtain expected absolute mean HAQ CFB and its 95% credible interval (95% CrI), and expected absolute probability of response and its 95% CrI, for ACR-50 and DAS28 < 2.6, for each biologic agent. For the relative efficacies as well as for the absolute responses, the point estimates reflect the most likely value for the parameter considered and the 95% credible intervals state that there is a 95% posterior probability that the parameter lies between the two values of the interval.

For the HAQ CFB analyses, the standard deviation was directly extracted from the publications where possible. When the standard deviation was not reported, it was estimated based on other statistics that allow calculation or estimation of the standard deviation (for example, confidence interval, standard error, t-value, *P*-value, F value). When no information about the uncertainty was available, the average of all the other standard deviations explicitly reported was imputed to the missing standard deviation, enabling integration of all the data available. The feasibility of the network meta-analysis was evaluated by means of a qualitative assessment of the comparability of the studies in terms of study design, treatments evaluated, patient population and quality of the network of studies. Differences across trials might act as effect modifiers and thereby potentially violate the similarity and consistency assumptions associated with network meta-analyses. Violation of these assumptions might introduce bias in the relative treatment effect estimates. Analyses were performed with WinBUGS 1.4 statistical software.

### Base-case and sensitivity analyses

The base case analysis of a network meta-analysis includes the broadest available evidence base corresponding to the question evaluated, under the condition of comparability for effect modifiers' characteristics. As the firm definition of such a case is often challenging, we pre-specified in the protocol that scenario analyses would be conducted along the base case, with an exact definition of these scenarios elaborated after the qualitative assessment of the included studies.

## Results

### Systematic review

**Figure 1:**
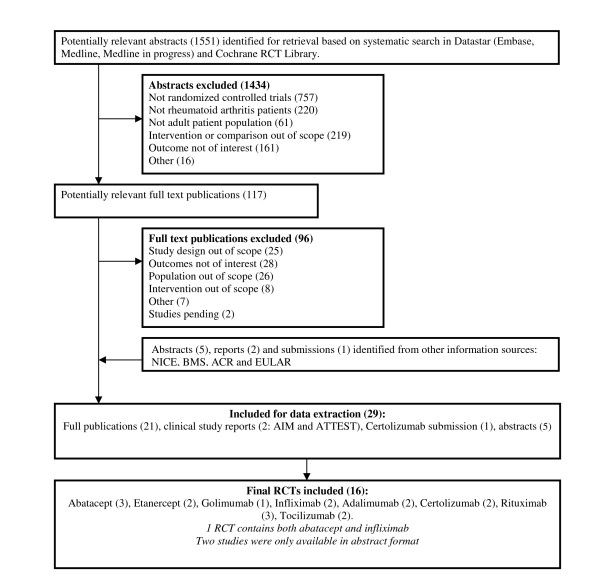
**Selection of included publications**. CFB, change from baseline; HAQ, Health Assessment Questionnaire; MTX, Methotrexate.

The systematic review identified 1,551 potentially relevant studies, of which 29 publications, including 2 Clinical Study Reports (CSRs), 1 NICE submission and 4 abstracts, were identified to be relevant. The study selection process is summarised in Figure 1. The 29 documents identified by the literature search included 16 individual studies for abatacept [14-18], adalimumab [24,25], certolizumab pegol [26-29], etanercept [30-32], golimumab [33,34], infliximab [15,35,36], rituximab [37-41] and tocilizumab [42-44]. Each comparison was supported by at least one pivotal trial, but not all trials reported findings for the HAQ CFB, the ACR-50 and DAS28 < 2.6 responders at either or both 24-week and 52-week follow-ups. All 16 included studies were randomised, double-blind and placebo-controlled.

### Study design and patient characteristics

As presented in Table 1, most studies were generally comparable in design, although differences were identified regarding patients not responding to treatment; the adalimumab studies included an early escape for non-responders [24] while the certolizumab pegol studies specifically withdrew patients who did not show an ACR20 response at weeks 12 and 14 [26-29]. Furthermore, the golimumab [33,34] and tocilizumab [43,44] studies provided rescue therapy for patients who did not achieve at least 20% improvement in both Tender Joint Count (TJC) and Swollen Joint Count (SJC) by week 16. The TEMPO trial [30,31] did not meet the inclusion criteria defined for the network meta-analyses; the study population was not comprised solely of patients diagnosed with RA showing an inadequate response to MTX. The SERENE study evaluating rituximab [37,38] and the LITHE study evaluating tocilizumab [42] were only publicly available in abstract format. Since no characteristics on design and patients were reported, no full evaluation of the comparability could be performed for these studies.

**Table 1:** Overview of trial designs

Trial (reference)	Compared interventions	Trial design; Patient population	Inclusion criteria	Endpoints	Study period
			*Exclusion criteria*		
*Abatacept studies*					
AIM trial [14,18]	PBO + MTX; ABA 10 mg/kg every four weeks + MTX	Phase 3 RCT; Active RA despite MTX	Met ACR criteria, > = 18 years, RA for > = 1 year, > = 10 SJC, > = 12 TJC, CRP > = 10.0 mg/L, MTX (≥ 15 mg/week) for ≥ 3 months with stable dose for 28 days prior to enrolment	ACR20 at six months; HAQ-DI (≥ 0.3); CFB in joint erosion score at one year	Nov '02 to Oct' 04; 52 weeks
			Positive tuberculin skin test		
Kremer *et al*. 2005, Kremer *et al*. 2003 [16,17]	PBO + MTX; ABA 2 mg/kg every four weeks + MTX; ABA 10 mg/kg every four weeks + MTX	Phase 3 RCT; Active RA despite MTX.	Met ACR criteria, > = 10 SJC, > = 12 TJC, CRP > 1 mg/dl, MTX (10 to 30 mg/week) for > = 6 months with stable dose for 28 days prior to enrolment	ACR20 at six months	Date n/s; 52 weeks + 6 months
			n/s		
ATTEST trial [15]	PBO + MTX; ABA 10 mg/kg every four weeks + MTX; INF 3 mg/kg every eight weeks + MTX	RCT, double-dummy, PBO and active (INF)-controlled; RA and MTX-IR	Met ACR criteria, > = 18 years, RA for > = 1 year, > 10 SJC, > 12 TJC, CRP > 1 mg/dl, MTX > 15 mg/week for > 3 months prior to randomisation, other DMARDs washed out	Reduction in DAS28 with abatacept vs placebo at six months	Feb '05 to June '06; 52 weeks PBO arm = PBO for six months followed by ABA
			Prior experience with abatacept or an other approved biologic RA therapy, failed on > 4 conventional DMARDs		
*Adalimumab studies*					
ARMADA [25]	PBO + MTX; ADA 20 mg every other week + MTX; ADA 40 mg every other week + MTX; ADA 80 mg every other week + MTX	RCT; Active RA despite MTX	Met ACR criteria; > = 18 years, > 9 TJC, > 6 SJC, MTX for > = 6 months and stable weekly dose for > = 4 weeks before enrolment, failed treatment with > = 1 DMARD besides MTX, but not > 4 DMARDs	ACR20	Date n/s; 24 weeks
			Prior anti-CD4 therapy or TNF-α antagonists, history of active listeriosis or mycobacterial infection, any major infection		
DE019 [24]	PBO + MTX; ADA 20 mg weekly + MTX; ADA 40 mg every other week + MTX	RCT; Active RA treated with MTX.	Met ACR criteria; > = 18 years, > = 9 TJC, > = 6 SJC, CRP > 1 mg/dl, either rheumatoid factor positivity or > = 1 joint erosion on radiographs of hands + feet, MTX for > = 3 months at stable dose of 12.5 to 25 mg/week for > = 4 weeks	ACR20 at week 24	Date n/s; 52 weeks
			Prior anti-CD4 therapy or TNF antagonists; history of: other active inflammatory arthritide, active listeriosis/mycobacterial infection, lymphoma/leukemia within five years; any major infection		
*Certolizumab Pegol studies*					
RAPID I [26,27,29]	PBO + MTX; CZP 200 mg every other week + MTX; CZP 400 mg every other week + MTX	Phase 3 RCT; Active RA with MTX-IR	> = 18 years, active RA for > = 6 months + < 15 years, > = 9 TJC + SJC with either ESR > = 30 m/hour or CRP > 15 mg/l, MTX for > = 6 months with a stable dosage of > = 10 mg/week for > = 2 months.	ACR20 at week 24; mean CFB in modified total Sharp score at week 52	Feb '05 to Oct' 06; 52 weeks. ACR20 non-responders at weeks 12 + 14 were withdrawn
			History of: tuberculosis, malignancy; PPD positive skin test; biologic therapy within 6 months, prior failure to respond to anti-TNF agent		
RAPID II [28,29]	PBO + MTX; CZP 200 mg every other week + MTX; CZP 400 mg every other week + MTX	Phase 3 RCT; Active RA despite > = 6 months of MTX	Met ACR criteria, > 18 years, RA > 6 months duration but < 15 years, MTX for > 6 months (stable dose > 10 mg/week for > 2 months at baseline)	ACR20 at week 24	June '05 to Sept' 06; 24 weeks ACR20 non-responders at weeks 12 + 14 were withdrawn
			Biologic agent in previous six months, severe reaction to biologic agents, no response to previous anti-TNF therapy, history of tuberculosis, PPD positive skin test		
*Etanercept studies*					
Weinblatt *et al*. 1999 [32]	PBO + MTX; ETN 25 mg 2x week + MTX	Double-blind, randomised; Active RA despite > = 6 months of MTX	Met ACR criteria, > = 18 years, > = 6 SJC + TJC, MTX for > = 6 months at stable dose of 15 to 25 mg/week for last 4 weeks, discontinued sulfasalazine + hydroxychloroquine > = 2 weeks + DMARDs other than MTX > = 4 weeks prior to study	ACR 20 at 24 weeks	Date n/s; 24 weeks
			n/s		
TEMPO [30,31]	PBO + MTX; ETN 25 mg 2x week; ETN 25 mg 2x week + MTX	Randomised, double-blind, parallel group study; RA patients with DMARD-IR	Met ACR criteria, > = 18 years, active disease for 6 months-20 years, > 10 SJC, > 12 painful joints, IR to > = 1 DMARD other than MTX, previous MTX (without toxic effects/lack of response), no MTX within 6 months of enrolment	ACR response (ACR-N) AUC for the first 24 weeks	Oct '00 to July' 01; 52 weeks
			Prior therapy with ETN or other TNF antagonists, immunosuppressive drugs within 6 months; investigational drug or biologic agent within 3 months, any other DMARDs or corticosteroid within 4 weeks, presence of relevant co morbidity		
*Golimumab studies*					
GO-FORWARD [33,34]	PBO + MTX; GOL 100 mg every four weeks; GOL 50 mg every four weeks + MTX; GOL 100 mg every four weeks + MTX	Phase 3 RCT; active RA despite MTX	Met ACR criteria, > 18 years, RA > = 3 months, tolerated stable MTX dose of 15-25 mg/week for > = 3 months prior to screening, > = 4 SJC & TJC, met the tuberculosis screening criteria	ACR20 at week 14 and improvement from baseline in HAQ-DI score at week 24.	Dec '05 to Sept' 07; 52 weeks. At week 16, patients < 20% CFB in TJC and SJC had medication adjusted
			Hypersensitivity to GOL, previous anti-TNF agent, RTX, natalizumab, cytotoxic agents, anakinra, DMARDs other than MTX, corticosteroids within four weeks, alefacept or efalizumab within three months.		
*Infliximab studies*					
ATTRACT [35,36]	PBO + MTX; INF 3 mg/kg every eight weeks + MTX; INF 3 mg/kg every four weeks + MTX; INF 10 mg/kg every eight weeks + MTX; INF 10 mg/kg every four weeks + MTX	International Phase 3 RCT; Active RA despite MTX	Met ACR criteria, > = 6 SJC + TJC, MTX for > = 3 months not stopped for > 2 weeks, MTX at stable dose > 12.5 mg/week for > = 4 weeks, oral corticosteroids or NSAIDs on stable dose for > = 4 weeks	ACR20 at week 30	Date n/s; 54 weeks
			DMARD (not MTX) or non-oral corticosteroids in four weeks before screening, alkylating agents, any other agent to reduce TNF, allergic to murine proteins, serious infections in previous three months, chronic infectious disease		
*Rituximab studies*					
DANCER [39,40]	PBO + MTX; RTX 500 mg x2 injections + MTX; RTX 1,000 mg x2 injections + MTX	Phase 2b international RCT, double-dummy; Active RA with DMARD-IR and MTX-IR.	Met ACR criteria, 19 to 79 years, RA > 6 months, MTX at 10 to 25 mg/week for > = 12 weeks before randomization, stable dose for last 4 weeks, > 8 SJC + TJC, either ECR > = 28 mm/hour or CRP > = 1.5 mg/dl, IR to 1 to 5 DMARDs (other than MTX) and/or biologic agents discontinued > = 4 weeks before randomization and INF, ADA, leflunomide > = 8 weeks before randomization	ACR20 for RF-positive patients at week 24	Date n/s; 24 weeks
			Significant systemic involvement secondary to RA, other illnesses or laboratory abnormalities, severe allergic or anaphylactic reactions to monoclonal antibodies, previous treatment with RTX or any lymphocyte-depleting therapies, recurrent significant infection		
			N/S		
Strand *et al*. 2006 [41]	PBO + MTX; RTX 1,000 mg x2 injections; RTX 1,000 mg x2 injections + cyclophosphamide; RTX 1,000 mg x2 injections + MTX	RCT; Active RA despite MTX	Met ACR criteria, > 21 years, MTX > = 10 mg/week, > = 8 SJC + TJC, CRP > = 15 mg/l and/or ESR > = 28 mm/h, and/or morning stiffness > 45 minutes, plasma rheumatoid factor level > 20 IU/ml	ACR 50 at week 24	Date n/s; 48 weeks
			Other autoimmune disease, ARA functional class IV disease, active rheumatoid vasculitis, history of systemic diseases associated with arthritis, chronic fatigue syndrome, serious and uncontrolled coexisting diseases		
SERENE [37,38]	PBO + MTX; RTX 500 mg x2 injections + MTX; RTX 1,000 mg x2 injections + MTX	Phase 3 RCT; Active RA with MTX-IR and naïve to prior biologic therapy	≥ 8 SJC + TJC, elevated CRP (≥ 0.6 mg/dL) and/or ESR (≥ 28 mm/h) despite MTX for ≥ 12 wks	ACR20 at week 24	Date n/s; 48 weeks
			N/S		
*Tocilizumab studies*					
OPTION [43,44]	PBO + MTX; TCZ 4 mg/kg every four weeks + MTX; TCZ 8 mg/kg every four weeks + MTX	Phase 3 RCT, parallel group; RA with MTX-IR	Met ACR criteria, adults, RA > 6 months, MTX-IR, > = 6 SJC, > = 8 TJC, CRP > 10 mg/L or ESR > = 28 mm/h, MTX for > = 12 weeks before start of study (stable dose of 10 to 25 mg/week for > = 8 weeks), discontinuation of other DMARDs: leflunomide > = 12 weeks, anakinra > = 1 week, etanercept > = 2 weeks, infliximab or adalimumab > = 8 weeks prior to start of study	ACR20 at 24 weeks	Date n/s; 24 weeks. At week 16, patients < 20% CFB in TJC and SJC were eligible for rescue therapy with TCZ 8 mg/kg
			other autoimmune diseases, significant systemic involvement secondary to RA, functional class IV RA, inflammatory joint disease other than RA, recurrent infections, active liver disease, anti-TNF agent failure		
			Not stated		
LITHE [42]	PBO + MTX; TCZ 4 mg/kg every four weeks + MTX; TCZ 8 mg/kg every four weeks + MTX	Phase 3 RCT, double-blind; RA with MTX-IR	N/S	CFB in Genant-modified Sharp score and AUC in the HAQ-DI at Week 52	two years A switch to blinded rescue treatment was available at weeks 16 and 28, if required.

ABA, abatacept; ADA, adalimumab; ARA, American Rheumatism Association; AUC, area under the curve; CFB, change from baseline; CZP, certolizumab pegol; DMARD-IR, inadequate response to DMARD; ETN, etanercept; GOL, golimumab; INF, infliximab; MTX-IR, inadequate response to MTX alone; n/s, not specified; PBO, placebo; PPD, purified protein derivative; RCT, randomised controlled trial; RF, rheumatoid factor; RTX, rituximab; SJC, swollen joint count; TCZ, tocilizumab; TJC, tender joint count; TNF, tumour necrosis factor

An overview of the baseline patient characteristics is provided in Table 2. All studies reported similar HAQ scores at baseline, except for the study by Kremer *et al*. 2005 [16], which presented a lower mean HAQ baseline value. This difference was likely to be due to the use of the modified HAQ (mHAQ) instead of the traditional Health Assessment Questionnaire Disability Index. Both instruments are strongly correlated with a Pearson correlation coefficient of 0.88 [45], so the difference in the instruments is assumed to have no impact on the relative treatment effect. For golimumab, the main publication [33] reported median and IQR data, instead of the expected means and SD, suggesting that data were not normally distributed. This study also included patients with lower swollen joint counts, a lower CRP level and shorter disease duration than most of the other studies in the network meta-analysis. Certolizumab pegol [26-29] and etanercept [30,31] included patients with a shorter disease duration compared to other identified trials. No information about the patient characteristics were provided for the SERENE and the LITHE studies.

**Table 2:** Overview of patient characteristics

Trial (reference)	Treatment arm	RF status (% positive)	Gender (% F)	Mean age (years)	Mean years since diagnosis	Mean n of prior DMARDs	% pts on NSAIDs	% pts on corticoid steriods	Mean TJC	Mean SJC	Mean pts pain^1 ^(100-mm VAS)	Mean pts GA ^2 ^(100-mm VAS)	Mean phs GA^3 ^(100-mm VAS)	Mean HAQ-DI	Mean CRP (mg/l)	Mean ESR (mm/h)
*Abatacept studies*																
AIM [14,18]	PBO + MTX; ABA 10 mg/kg every four weeks + MTX	81.8	77.8	51.5	8.5	1.3	85.5	72.1	31.0	21.4	63.3	62.7	68.0	1.70	33	nr
		78.5	81.7	50.4	8.9	1.2	82.6	68.5	32.3	22.1	65.9	62.8	67.4	1.70	28	
Kremer *et al*. 2005, Kremer *et al*. 2003 [16,17]	PBO + MTX	90.0	66.0	54.7	8.9	21%	nr	67.2	29.2	21.8	65.2	62.8	63.3	1.00	32	nr
	ABA 2 mg/kg every four weeks + MTX	90.0	63.0	54.4	9.7	18.1%		67.6	28.2	20.2	64.5	59.4	61.0	1.00	32	
	ABA 10 mg/kg every four weeks + MTX	99.0	75.0	55.8	9.7	16.5%		60.0	30.8	21.3	62.1	60.1	62.1	1.00	29	
ATTEST [15]	ABA 10 mg/kg every four weeks + MTX	87.2	83.3	49.0	7.9	1.7	85.3	75.6	31.6	21.3	nr	nr	nr	1.80	31	49.4
	PBO + MTX	77.3	87.3	49.4	8.4	1.8	84.5	70.0	30.3	20.1				1.80	27	47.0
	INF 3 mg/kg every eight weeks +MTX	84.8	82.4	49.1	7.3	1.7	86.1	71.5	31.7	20.3				1.70	33	47.8
*Adalimumab studies*																
ARMADA [25]	PBO + MTX	mean IU/liter +- SD reported	82.3	56.0	11.1	3.0	nr	nr	28.7	16.9	57.2	58.0	58.9	1.64	31	nr
	ADA 20 mg every other week + MTX		75.4	53.5	13.1	3.0			28.5	17.6	55.1	57.6	60.5	1.52	28	
	Adalimumab 40 mg every other week + MTX		74.6	57.2	12.2	2.9			28.0	17.3	53.0	56.9	58.7	1.55	21	
	ADA 80 mg every other week + MTX		75.3	55.5	12.8	3.1			30.3	17.0	55.0	58.8	62.6	1.55	28	
DE019 [24]	ADA 40 mg every other week + MTX	81.6	76.3	56.1	11.0	2.4	nr	nr	27.3	19.3	55.9	52.7	62.0	1.45	18	nr
	ADA 20 mg weekly + MTX	81.2	75.5	57.3	11.0	2.4			27.9	19.6	55.2	51.9	61.6	1.44	14	
	PBO + MTX	89.5	73.0	56.1	10.9	2.4			28.1	19.0	56.3	54.3	61.3	1.48	18	
*Certolizumab Pegol studies*																
RAPID I [26,27,29]	PBO + MTX	82.8	83.9	52.2	6.2	1.4	nr	nr	29.8	21.2	nr	nr	nr	1.70	16	45.0
	CZP 200 mg every other week + MTX	79.6	82.4	51.4	6.1	1.3			30.8	21.7				1.70	16	43.5
	CZP 400 mg every other week + MTX	83.6	83.6	52.4	6.2	1.3			31.1	21.5				1.70	14	42.5
RAPID II [28,29]	PBO + MTX	78.2	84.3	51.5	5.6	1.2	nr	nr	30.4	21.9	59.9	59.9	65.7	1.60	14	40.8
	CZP 200 mg every other week + MTX	77.5	83.7	52.2	6.1	1.2			30.1	20.5	61.8	62.4	64.3	1.60	14	43.7
	CZP 400 mg every other week + MTX	75.5	78.0	51.9	6.5	1.3			30.0	21.0	60.5	61.1	62.8	1.60	13	39.1
*Etanercept studies*																
Weinblatt *et al*. 1999 [32]	PBO + MTX	90.0	73.0	53.0	13.0	2.8	80.0	70.0	28.0	17.0	56.0	60.0	65.0	1.50	26	36.0
	ETN 25 mg twice weekly + MTX	84.0	90.0	48.0	13.0	2.7	75.0	53.0	28.0	20.0	50.0	60.0	60.0	1.50	22	25.0
TEMPO [30,31]	PBO + MTX	71.0	79.0	53.0	6.8	2.3	86.0	64.0	33.1	22.6	nr	nr	nr	nr	26	nr
	ETN 25 mg twice weekly	75.0	77.0	53.2	6.3	2.3	88.0	57.0	35.0	23.0					32	
	ETN 25 mg twice weekly + MTX	76.0	74.0	52.5	6.8	2.3	88.0	62.0	34.2	22.1					30	
*Golimumab studies*																
GO-FORWARD [33,34]	PBO + MTX	81.2	82.0	52.0	6.5	70.7^4^	nr	nr	21.0	12.0	57.0	53.0	56.5	1.25	8	nr
	GOL 100 mg every 4 weeks	83.5	78.9	51.0	5.9	75.9			22.0	11.0	60.0	56.0	58.0	1.38	9	
	GOL 50 mg every 4 weeks + MTX	86.5	80.9	52.0	4.5	78.7			26.0	13.0	61.0	60.0	61.0	1.38	10	
	GOL 100 mg every four weeks + MTX	84.3	80.9	50.0	6.7	75.3			23.0	12.0	64.0	59.0	61.0	1.38	9	
*Infliximab studies*																
ATTRACT [35,36]	PBO + MTX	77.0	80.0	51.0	8.9	2.5	72.0	64.0	24.0	19.0	67.0	62.0	65.0	1.80	30	nr
	INF 3 mg/kg every eight weeks +MTX	84.0	81.0	56.0	8.4	2.8	79.0	63.0	32.0	19.0	70.0	66.0	61.0	1.80	31	
	INF 3 mg/kg every four weeks +MTX	80.0	77.0	51.0	7.2	2.6	76.0	53.0	31.0	20.0	69.0	57.0	62.0	1.80	20	
	INF 10 mg/kg every eight weeks +MTX	82.0	77.0	55.0	9.0	2.5	77.0	57.0	30.0	20.0	67.0	64.0	64.0	1.80	25	
	INF 10 mg/kg every four weeks +MTX	82.0	73.0	52.0	8.7	2.5	68.0	65.0	35.0	23.0	66.0	60.0	60.0	1.50	24	
*Rituximab studies*																
DANCER [39,40]	PBO + MTX	100 (efficacy analyses)	80.0	51.1	9.3	2.2	nr	nr	35.0	21.0	nr	nr	nr	1.70	33	40.0
	RTX 500 mg * two injections + MTX	100	83.0	51.4	11.1	2.5			33.0	22.0				1.80	32	45.0
	RTX 1,000 mg * two injections + MTX	100	80.0	51.1	10.8	2.5			32.0	22.0				1.70	30	41.0
Edwards *et al*. 2004 [41]	PBO + MTX	100	80.0	54.0	11.0	2.6	nr	nr	32.0	19.0	nr	nr	nr	nr	32	52.0
	RTX 1,000 mg * two injections	100	73.0	54.0	9.0	2.5			34.0	21.0					26	47.0
	RTX 1,000 mg * two injections +cyclophosphamide	100	83.0	53.0	10.0	2.6			33.0	19.0					40	55.0
	RTX 1,000 mg * two injections + MTX	100	75.0	54.0	12.0	2.5			32.0	23.0					29	53.0
*Tocilizumab studies*																
OPTION [43,44]	PBO + MTX	78.0	78.0	50.6	7.8	1.7	68.0	nr	32.8	20.7	57.3	63.6	63.7	1.50	24	49.7
	TCZ 4 mg/kg every four weeks + MTX	83.0	82.0	51.4	7.4	1.5	68.0		33.2	20.0	60.7	65.6	63.6	1.60	28	49.2
	TCZ 8 mg/kg every four weeks + MTX	71.0	85.0	50.8	7.5	1.5	66.0		31.9	19.5	59.9	64.8	64.0	1.60	26	51.2
LITHE [42]	PBO + MTX	nr	nr	nr	nr	nr	nr	nr	nr	nr	nr	nr	nr	1.5	nr	nr
	TCZ 4 mg/kg every four weeks + MTX													1.5		
	TCZ 8 mg/kg every four weeks + MTX													1.5		

The treatment arms in grey were not used in the network meta-analysis but are included as part of the trials.

^1^Patients assessment of pain (Pts Pain); ^2^Patients global assessment of disease activity (Pts GA); ^3^Physician global assessment of disease activity (Phs GA); ^4^% Patients with previous use of DMARD other than MTX. ABA, abatacept; ADA, adalimumab; CRP, C-reactive protein; CZP, certolizumab pegol; DMARD, Disease-modifying anti-rheumatic drug; ESR, erythrocyte sedimentation rate; ETN, etanercept; GA, global assessment; GOL, golimumab; HAQ, health assessment questionnaire; INF, infliximab; MTX, methotrexate; nr, not recorded; NSAID, non-steroidal anti-inflammatory drug; PBO, placebo; Pts, patients; RF, rheumatoid factor; RTX, rituximab; SJC, swollen joint count; TCZ, tocilizumab; TJC, tender joint count;

No data for patient characteristics were available for the SERENE study.

The reported data for HAQ change from baseline at 24 and 52 weeks are presented in Table 3. A network meta-analysis was performed including 14 studies in the base case. Etanercept was evaluated in only two trials: Weinblatt 1999 [32] and TEMPO trial. As Weinblatt 1999 is a relatively small study (89 patients included), it was decided to retain the TEMPO trial in the base case analysis and to evaluate the impact of exclusion in a scenario analysis. It was decided to evaluate the inclusion of the SERENE and LITHE studies in sensitivity analyses in anticipation of the full text publications. Since comparability of the study design characteristics and the patients' characteristics could not be performed, the results need to be interpreted with this limitation in mind. Other observed differences between trials could not be explored in scenario analyses, as excluding these studies would have removed the treatments from the analysis.

**Table 3:** Reported data for HAQ CFB, ACR-50 and DAS28 < 2.6 at 24 and 52 weeks

Trial	N	Mean HAQ CFB at 24 weeks (SD)	Mean HAQ CFB at 52 weeks (SD)	ACR-50 r at 24 weeks	ACR-50 r at 52 weeks	DAS28 < 2.6 r at 24 weeks	DAS28 < 2.6 r at 52 weeks
***Placebo + MTX***							
AIM [14,18]	219	-0.40 (0.59)	-0.37 (0.59)	37	40	6	4
Kremer *et al*. 2005, Kremer *et al*. 2003 [16,17]	119	-0.14 (0.49*)	-0.10 (0.83*)	14	24	11	12
ATTEST [15]	110	-0.29 (0.22)		22		3	
ARMADA [25]	62	-0.27 (0.57)		5			
DE019 [24]	200	-0.24 (0.52)	-0.25 (0.56)	19	19		
RAPID I [26,27,29]	199	-0.17 (0.56)	-0.18 (0.56)	15	15		
RAPID II [28,29]	127	-0.14 (0.45)		4		1	
Weinblatt *et al*. 1999 [32]	30	-0.40 (0.49*)		1			
TEMPO [30,31]	228	-0.63 (1.08*)	-0.63 (1.41*)	92	91	31	39
GO-FORWARD [33,34]	133	-0.13 (0.58)		18		8	
ATTRACT [35,36]	88	-0.19 (0.49*)	-0.17 (0.60*)		8		
DANCER [39,40]	122	-0.28 (0.50)		16			
Strand *et al*. 2006 [41]	40	-0.40 (0.62*)	-0.30 (0.64*)	5	2		
SERENE [37,38]	172	-0.19 (0.56*)	-0.19^† ^(0.60*)	15	15	3	3
OPTION [43,44]	204	-0.34 (0.83*)		22		1	
LITHE [42]	393				39		12
**Abatacept + MTX**							
AIM [14,18]	433	-0.59 (0.62)	-0.66 (0.62)	173	209	64	103
Kremer *et al*. 2005, Kremer *et al*. 2003 [16,17]	115	-0.42 (0.49*)	-0.47 (0.83*)	42	48	30	40
ATTEST [15]	156	-0.68 (0.22)	-0.67 (0.62)	63	71	18	29
**Adalimumab + MTX**							
ARMADA [25]	67	-0.62 (0.63)		37			
DE019 [24]	207	-0.56 (0.52)	-0.59 (0.57)	81	86		
**Certolizumab + MTX**							
RAPID I [26,27,29]	393	-0.58 (0.59)	-0.60 (0.59)	146	147		
RAPID II [28,29]	246	-0.50 (0.47)		80		23	
**Etanercept + MTX**							
Weinblatt *et al*. 1999 [32]	59	-0.70 (0.49*)		23			
TEMPO [30,31]	231	-0.89 (1.08*)	-0.97 (1.41*)	136	180	70	88
**Golimumab + MTX**							
GO-FORWARD [33,34]	89	-0.47 (0.55)		33		18	
**Infliximab + MTX**							
ATTEST [15]	165	-0.53 (0.29)	-0.59 (0.64)	61	60	21	20
ATTRACT [35,36]	86	-0.31 (0.49*)	-0.32 (0.60*)		21		
**Rituximab + MTX**							
DANCER [39,40]	122	-0.49 (0.55)		41			
Strand *et al*. 2006 [41]	40	-0.60 (0.92*)	-0.60 (0.88*)	17	14		
SERENE [37,38]	170	-0.42 (0.54*)	-0.47 (0.60*)	44	61	15	19
**Tocilizumab + MTX**							
OPTION [43,44]	205	-0.55 (0.82*)		90		47	
LITHE [42]	398				145		127

*Standard deviation (SD) was estimated.

^†^No placebo value was available at 52 weeks for placebo so the values have been assumed equal to the values for 24 weeks.

ABA, abatacept; ADA, adalimumab; CFB, change from baseline; CZP, certolizumab pegol; ETN, etanercept; GOL, golimumab; INF, infliximab; N, number of patients in trial; PBO, placebo; r, number of responders; RTX, rituximab; TCZ, tocilizumab

### Network Meta-analysis results (Tables 4 and 5)

#### HAQ change from baseline at 24 and 52 weeks

At 24 weeks, all biologic agents in combination with MTX were found to be more effective than placebo in combination with MTX in improving functional status (HAQ CFB). Small numerical differences were observed in favor of abatacept over etanercept, infliximab, rituximab and tocilizumab. The adjusted absolute mean HAQ change from baseline varied between -0.48 and -0.67 for the biologic agents considered. Abatacept showed comparable efficacy compared to other biologics at 24 weeks (absolute mean HAQ change from baseline of -0.58). At 52 weeks, the findings were in line with those at 24 weeks. All biologics demonstrated a higher reduction in HAQ score compared to placebo and a comparable efficacy relative to the other biologic agents, with a trend in favor of abatacept over infliximab (-0.11, 95% CrI: -0.22; 0.01)).

**Table 4:** Relative efficacy versus abatacept + MTX at 24/26 and 48/54 weeks

Treatment effect relative to Abatacept + MTX	Difference in mean HAQ CFB at 24/26 weeks (95% CrL)*	Difference in mean HAQ CFB at 48/54 weeks (95% CrL)**	OR for ACR-50 at 24/26 weeks (95% CrL)*	OR for ACR-50 at 48/54 weeks (95% CrL)**	OR for DAS28 < 2.6 at 24/26 weeks (95% CrL)*	OR for DAS28 < 2.6 at 48/54 weeks (95% CrL)*
Placebo + MTX	-0.30 (-0.41; -0.18)	-0.29 (-0.38; -0.21)	3.37 (1.49; 8.06)	3.84 (2.84; 5.26)	4.77 (1.60; 15.78)	8.82 (1.50; 57.83)
Adalimumab + MTX	0.03 (-0.16; 0.24)	0.05 (-0.09; 0.18)	0.40 (0.09; 1.50)	0.56 (0.29; 1.03)		
Certolizumab Pegol + MTX	0.08 (-0.09; 0.28)	0.13 (-0.00; 0.26)	0.35 (0.08; 1.33)	0.51 (0.26; 0.96)	0.26 (0.01; 3.90)	
Etanercept + MTX	-0.02 (-0.24; 0.21)	0.05 (-0.22; 0.32)	1.05 (0.17; 3.24)	0.72 (0.43; 1.19)	1.69 (0.21; 15.80)	2.94 (0.14; 67.12)
Golimumab + MTX	0.04 (-0.21; 0.30)		0.87 (0.16; 5.15)		1.18 (0.13; 11.66)	
Infliximab + MTX	-0.11 (-0.29; 0.08)	-0.11 (-0.22; 0.01)	1.31 (0.27; 7.61)	1.40 (0.93; 2.10)	0.88 (0.09; 7.76)	1.68 (0.14; 21.23)
Rituximab + MTX	-0.09 (-0.31; 0.14)	0.01 (-0.34; 0.35)	0.85 (0.20; 3.47)	0.31 (0.04; 1.37)		
Tocilizumab + MTX	-0.09 (-0.35; 0.18)		0.51 (0.10; 2.88)		0.05 (0.00; 0.79)	

Note: for HAQ CFB, negative values indicate a trend towards a clinical benefit for abatacept. For ACR-50 and DAS28 < 2.6, an OR > 1 indicates a trend towards a clinical benefit for abatacept.

* Results based on a random effects model. ** Results based on a fixed effects model.

OR, odds ratio; 95% CrL, 95% credible limits

**Table 5:** Adjusted absolute efficacy for biologic DMARDS + MTX at 24/26 and 48/54 weeks

Treatments relative to effect	Absolute HAQ CFB at 24/26 weeks (95% CrL)*	Absolute HAQ CFB at 48/54 weeks (95% CrL)**	Proportion (%) of patients with ACR-50 at 24/26 weeks (95% CrL) *	Proportion (%) of patients with ACR-50 at 48/54 weeks (95% CrL)**	Proportion (%) of patients with DAS28 < 2.6 at 24/26 weeks (95% CrL)*	Proportion (%) of patients with DAS28 < 2.6 at 48/54 weeks (95% CrL)*
Placebo + MTX	-0.29 (-0.31; -0.26)	-0.29 (-0.34; -0.24)	11.9% (9.7%; 14.0%)	12.5% (9.4%; 15.5%)	2.6% (1.4%; 4.1%)	7.0% (4.7%; 9.8%)
Adalimumab + MTX	-0.61 (-0.77; -0.46)	-0.63 (-0.74; -0.51)	53.5% (28.0%; 77.9%)	49.5% (35.9%; 63.5%)		
Certolizumab Pegol + MTX	-0.67 (-0.82; -0.53)	-0.71 (-0.81; -0.61)	57.3% (31.2%; 79.9%)	51.7% (38.1%; 66.1%)	33.4% (4.4%; 90.0%)	
Etanercept + MTX	-0.56 (-0.75; -0.38)	-0.63 (-0.87; -0.39)	30.7% (15.6%; 65.2%)	43.2% (31.8%; 54.9%)	6.9% (1.0; 31.5%)	18.7% (1.8%; 73.3%)
Golimumab + MTX	-0.63 (-0.86; -0.39)		34.6% (9.7%; 69.2%)		9.6% (1.4%; 42.4%)	
Infliximab + MTX	-0.48 (-0.62; -0.33)	-0.48 (-0.59; -0.36)	26.0% (6.6%; 57.4%)	28.1% (19.4%; 38.3%)	12.6% (1.9%; 53.3%)	28.8% (1.9%; 89.5%)
Rituximab + MTX	-0.49 (-0.68; -0.31)	-0.59 (-0.91; -0.27)	35.3% (14.8%; 61.9%)	64.1% (32.4%; 91.3%)		
Tocilizumab + MTX	-0.49 (-0.73; -0.26)		47.5% (16.1%; 78.6%)		71.0% (19.0%; 98.9%)	
Abatacept + MTX	-0.58 (-0.70; -0.46)	-0.58 (-0.66; -0.50)	31.7% (15.9%; 50.6%)	35.4% (27.3%; 43.3%)	11.3% (3.7%; 28.8%)	40.2% (10.4%; 80.3%)

Note: For HAQ CFB, negative values indicate improvement.

* Results based on a random effects model. ** Results based on a fixed effects model.

95% CrL, 95% credible limits

**Figure 2:**
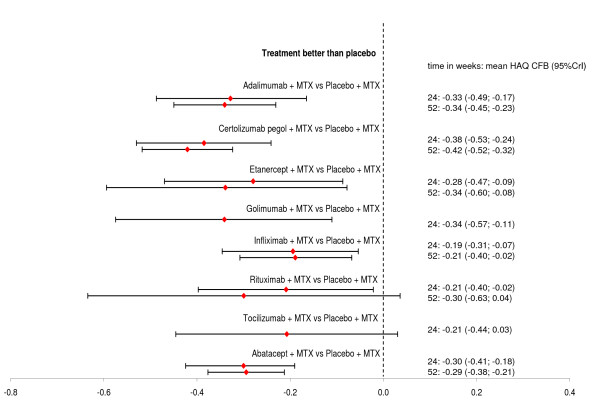
**Relative HAQ CFB of each biologic versus placebo**. CFB, change from baseline; HAQ, Health Assessment Questionnaire; MT,: Methotrexate.

Figure 2 illustrates each pairwise relative efficacy of all biologic agents compared to placebo at 24 and 52 weeks.

#### ACR-50 response rates at 24 and 52 weeks

At 24 weeks, all biologic agents demonstrated a higher proportion of ACR-50 responders than placebo, and abatacept is expected to demonstrate comparable ACR-50 response rates as to the other biologic agents. The expected proportion of patients with ACR-50 response was estimated to be 31.7% (95% CrI: 15.9%; 50.6%) for abatacept, which is higher than those for placebo (11.9%, 95% CrI: 9.7%; 14.0%) and comparable to the other biologic agents (expected proportions between 26.0% and 57.3%). At 52 weeks, abatacept is expected to result in a higher proportion of responders than placebo and comparable response rates to other biologic agents except for certolizumab pegol (OR:0.51, 95% CrI: 0.26; 0.96) although these results need to be interpreted with caution due to the earlier described difference in trial design. The expected proportion of ACR-50 responders for abatacept was slightly higher (35.4%, 95% CrI: 27.3%; 43.3%) than those at 24 weeks.

#### DAS28 defined remission (< 2.6) at 24 and 52 weeks

At 24 weeks, no data were available for adalimumab and rituximab. Abatacept was found to result in more patients with DAS28 defined remission than placebo, with an OR of 4.77 (95% CrI: 1.60; 15.78). Abatacept is expected to be less efficacious than tocilizumab, but showed findings comparable to all other biologic agents. The expected proportion of patients under remission at 24 weeks amongst the biologics ranged from 6.9% to 71.0%. At 52 weeks, data were only available for infliximab, etanercept and abatacept. Abatacept was found to result in more DAS28 responders than the placebo and in comparable remission rates compared to infliximab and etanercept. The expected proportion of patients under remission at 52 weeks for abatacept was higher (40.2%, 95% CrI: 10.4%; 80.3%) than at 24 weeks.

### Sensitivity analyses

The TEMPO trial was included in the base case analysis as it was the pivotal trial for etanercept in this patient population. However, the TEMPO trial included a DMARD-IR population rather than a MTX-IR population as included in the other trials and also showed high observed response rates in the control group, which is substantially different from observed findings in other studies. The patient selection criteria in the TEMPO trials allowed for inclusion of patients not treated with MTX, potentially explaining the high response rate observed in the control arm. Removing the TEMPO trial did not significantly impact on the findings for the mean HAQ CFB at 24 weeks: abatacept was found to be comparable in efficacy to all biologics, including etanercept (difference in HAQ CFB vs. etanercept: 0.00 (95% CrI: -0.32; 0.33)). However, excluding the TEMPO trial from the ACR-50 analysis at 24 weeks did have an impact on the results. By excluding this trial the heterogeneity was reduced and goodness of fit statistics suggested the use of a fixed effects model. This resulted in smaller credible intervals around the point estimates. As a result, abatacept was found to be more efficacious than placebo (OR: 3.31, 95% CrI: 2.47; 4.48) although less efficacious than certolizumab pegol (OR: 0.37, 95% CrI: 0.20; 0.64), adalimumab (OR: 0.43, 95% CrI: 0.24; 0.75), etanercept (OR: 0.12, 95% CrI: 0.00; 0.82) and tocilizumab (OR: 0.50, 95% CrI: 0.27; 0.91). Abatacept showed comparable efficacy to golimumab, infliximab and rituximab. Differences in trial design that might explain these findings are described in the discussion section.

The TEMPO trial did not report HAQ data at 52 weeks and was the only trial reporting ACR-50 data for etanercept at 52 weeks, and the only trial reporting DAS28 defined remission data for etanercept at both follow-ups, limiting the evaluation of excluding TEMPO on these endpoints.

In the base case analysis all randomised patients were included for the AIM study, although patients included from one site were excluded from the efficacy analyses because of protocol violations. Its impact on the findings was evaluated in a sensitivity analysis and did not change the relative efficacy of abatacept to other biologic agents (data not reported).

Including the data for the SERENE [37,38] study, evaluating rituximab, and the LITHE [42] study, evaluating tocilizumab did not substantially impact the results. The SERENE study presents HAQ CFB, ACR-50 and DAS28 < 2.6 data at both follow-ups. The LITHE study only reports ACR-50 and DAS28 defined remission response rates at 52 weeks. Abatacept showed comparable efficacy versus rituximab at 24 weeks: (mean difference in HAQ CFB: -0.08 95% CrI: -0.24; 0.10), ACR-50 (OR: 0.87, 95% CrI: 0.31; 2.30), DAS28 < 2.6 (OR: 0.80, 95% CrI: 0.07; 8.20), and at 52 weeks (mean difference in HAQ CFB: -0.01, 95% CrI: -0.36; 0.31), ACR-50 (OR: 0.55, 95% CrI: 0.13; 1.78), DAS28 < 2.6 (OR: 1.09, 95% CrI: 0.04; 30.72). Abatacept demonstrated comparable efficacy versus tocilizumab at 52 weeks (ACR-50 (OR: 0.73, 95% CrI: 0.17; 3.12), DAS28 < 2.6 (OR: 0.58, 95% CrI: 0.03; 14.23)).

## Discussion

A network meta-analysis based on a systematic review of the literature was performed to estimate the relative efficacy of abatacept compared with other relevant biologic DMARDs in the treatment of RA patients with insufficient response to MTX. The results of the network meta-analysis showed that abatacept is expected to be more efficacious than placebo and show comparable efficacy relative to the other biologic DMARDs in combination with MTX. The primary outcome in the present study was the reduction in functional status as measured by the HAQ score, which is commonly used in economic modeling of RA since this can be translated into required utility values by means of published algorithms. Also, the clinically relevant endpoints ACR-50 and DAS28-defined remission (< 2.6) at 24 weeks and 52 weeks were analysed. Not all trials reported findings on all evaluated endpoints. The decision was made to include all available data leading to differences in evidence used across endpoints.

The analysis of DAS28-defined remission at 24 weeks showed comparable findings to other biologic agents for abatacept, except in the case of tocilizumab. It should be noted that tocilizumab, due to its mechanism of action, has a direct effect on the CRP-level and, therefore, is expected to show more efficacy on this endpoint. Also, a low number of patients in remission were observed in the placebo arms across the trials, making the indirect treatment comparison susceptible to small differences in the placebo arms. As a consequence, results should be interpreted cautiously.

Although the TEMPO trial included different patients, it was decided to include this study based on the fact that TEMPO is the pivotal trial for etanercept. Had TEMPO been excluded from the base case, data for etanercept would have been based solely on a relatively old and small trial (89 patients) by Weinblatt (1999) [32], potentially biasing the findings in favor of etanercept.

Other limitations in comparability of study and patient characteristics were observed with the adalimumab, golimumab and certolizumab pegol trials. The adalimumab studies included an early escape for non-responders [24] while the certolizumab pegol studies specifically withdrew patients who did not show an ACR20 response at weeks 12 and 14 [26-29]. Furthermore, the golimumab [33,34] and tocilizumab [43,44] studies provided rescue therapy for patients who did not achieve at least 20% improvement in both Tender Joint Count and Swollen Joint Count by week 16. The impact associated with the adalimumab, golimumab and certolizumab pegol studies was not explored in scenario analyses, as excluding these studies would have removed the treatments from the analysis and this would not have provided additional information. Furthermore, there is currently no consensus on how to correct for these differences in trial design.

All patients in the studies received methotrexate in the trial, independent of whether they were assigned to the placebo or intervention arm. The fact that optimal methotrexate dosing was decided by the investigator and that the trials differ in specification of minimal methotrexate dose may result in differences across the trials. In turn, this may have had interaction with the observed effect for the biologic agents and, therefore, is potentially introducing bias in the analysis. Unfortunately, we were unable to correct for this since methotrexate details are lacking.

A recent network meta-analysis of tocilizumab and other biologic agents in patients who have an inadequate response to conventional DMARDs or MTX [46] suggests that tocilizumab has a better overall response than TNF-α inhibitors and abatacept, whereas our analyses suggest comparable efficacy. The apparent distinction may be attributable to differences in the selection criteria for relevant studies (MTX vs. conventional DMARDs background treatment) and, therefore, the evidence base and analysis techniques (fixed versus random approaches). The TOWARD trial [47] was not included in our analyses and no data on HAQ score were available for the LITHE trial. Similarly, despite important differences in the study selection process, the Cochrane collaboration found that abatacept, adalimumab, etanercept, infliximab and rituximab showed comparable efficacy in patients with RA [48]. The Cochrane collaboration also performed a network meta-analysis on the safety of the biologic agents [49]. This study revealed that abatacept was associated with a significantly lower risk of serious adverse events compared to most other biologics and was significantly less likely than infliximab and tocilizumab to be associated with serious infections. When comparing different treatments, safety should always be considered in addition to efficacy. In our study no evaluation of safety was performed as this would have required a different search strategy. Finally, a systematic review [50] followed by several meta-analyses of nine biological DMARDS (including abatacept) vs. placebo was performed and used to inform the EULAR recommendation [51]. In this publication, all biological DMARDs + MTX combinations were found to be more efficacious than placebo + MTX in the treatment of patients with an inadequate response to MTX.

## Conclusions

Currently it is not possible to predict, on an individual basis, which patient will respond to a particular therapy. This is a significant unmet need which is the goal of much research effort. In the absence of reliable biomarkers on which to base individual treatment decisions, it is important that patients have access to the full range of biologic therapeutics with different mechanisms of action and proven efficacy. This network meta-analysis strongly suggests that abatacept in combination with MTX is superior to placebo and is comparable to other biologic DMARDs for the reduction in disability (HAQ CFB) of RA for at least a year of treatment in patients with active disease despite previous treatment with MTX. Abatacept in combination with MTX is also expected to be superior to placebo and comparable to all other biologic agents for ACR-50, with the exception of certolizumab pegol at 52 weeks, although this needs to be interpreted with caution due to the earlier described difference in trial design, and comparable efficacy in DAS28 defined remission at 24 weeks (except for tocilizumab, which can be explained by the causal relation with the CRP level).

Based on its unique mechanism of action, relative efficacy and clinical trial safety profile [14-18], abatacept is a suitable alternative to currently licensed biologic DMARDs, meaning that abatacept in combination with MTX should be available to patients with RA, which is refractory to MTX alone.

## Abbreviations

ACR50 (20): American College of Rheumatology 50% (20%) improvement criteria; CFB: change from baseline; CrI: credible intervals; CRP: C-reactive protein; CSRs: Clinical Study Reports; DAS: disease activity score; DMARD: disease-modifying antirheumatic drug; EULAR: European League Against Rheumatism; HAQ: Health Assessment Questionnaire; MsSH: Medline Subject Headings; MTX: methotrexate; NICE: National Institute of Health and Clinical Excellence; OR: odds ratio; RA: rheumatoid arthritis; RCTs: randomised controlled trials; SJC: Swollen Joint Count; TJC: Tender Joint Count; TNF: tumour necrosis factor.

## Competing interests

PG and GB served as consultants to BMS and Roche in the field of rheumatoid arthritis and to other pharmaceutical companies for other indications. PCT has received research grants from Merck, UCB, AstraZeneca, GlaxoSmithKline and Roche. He has been an advisor for Abbott, Bristol-Myers Squibb, Roche, Schering-Plough, Wyeth and UCB. As head of Musculoskeletal Statistics Unit, at The Parker Institute, RC has received consulting fees, honoraria, research or institutional support, educational grants, equipment, services or expenses from: Abbott, Amgen, Astellas Pharma, Axellus, Bristol-Myers Squibb, Cambridge Nutritional Foods, Centocor, Dansk Droge, DSM Nutritional Products, Expanscience, Genentech, Hyben Vital, Hypo-Safe, MSD, MundiPharma, NorPharma, NutriCare, Pharmavie, Pfizer, Roche, Sanofi-Aventis, the Scandinavian Clinical of Nutrition, UCB, Wyeth. CP, LP, ML and PD declare they have no competing interests.

## Authors' contributions

All authors were involved in the conception of study design, analysis and interpretation of data, drafting the article or revising it critically for important intellectual content, and all authors approved the final version to be published.
